# Improved benchmarks for computational motif discovery

**DOI:** 10.1186/1471-2105-8-193

**Published:** 2007-06-08

**Authors:** Geir Kjetil Sandve, Osman Abul, Vegard Walseng, Finn Drabløs

**Affiliations:** 1 Department of Computer and Information Science, Norwegian University of Science and Technology (NTNU), Trondheim, Norway; 2 Department of Computer Engineering, TOBB University of Economics and Technology, Ankara, Turkey; 3 Department of Cancer Research and Molecular Medicine, Norwegian University of Science and Technology (NTNU), Trondheim, Norway

## Abstract

**Background:**

An important step in annotation of sequenced genomes is the identification of transcription factor binding sites. More than a hundred different computational methods have been proposed, and it is difficult to make an informed choice. Therefore, robust assessment of motif discovery methods becomes important, both for validation of existing tools and for identification of promising directions for future research.

**Results:**

We use a machine learning perspective to analyze collections of transcription factors with known binding sites. Algorithms are presented for finding position weight matrices (PWMs), IUPAC-type motifs and mismatch motifs with optimal discrimination of binding sites from remaining sequence. We show that for many data sets in a recently proposed benchmark suite for motif discovery, none of the common motif models can accurately discriminate the binding sites from remaining sequence. This may obscure the distinction between the potential performance of the motif discovery tool itself versus the intrinsic complexity of the problem we are trying to solve. Synthetic data sets may avoid this problem, but we show on some previously proposed benchmarks that there may be a strong bias towards a presupposed motif model. We also propose a new approach to benchmark data set construction. This approach is based on collections of binding site fragments that are ranked according to the optimal level of discrimination achieved with our algorithms. This allows us to select subsets with specific properties. We present one benchmark suite with data sets that allow good discrimination between positive and negative instances with the common motif models. These data sets are suitable for evaluating algorithms for motif discovery that rely on these models. We present another benchmark suite where PWM, IUPAC and mismatch motif models are not able to discriminate reliably between positive and negative instances. This suite could be used for evaluating more powerful motif models.

**Conclusion:**

Our improved benchmark suites have been designed to differentiate between the performance of motif discovery algorithms and the power of motif models. We provide a web server where users can download our benchmark suites, submit predictions and visualize scores on the benchmarks.

## Background

Computational discovery of motifs in biological sequences is an important challenge. It has in recent years attracted much research interest, resulting in more than a hundred different tools for motif discovery [1]. A motif discovery method has three important elements: a motif model that can capture the similarities of a diverse set of binding sites for the same transcription factor, an objective function defining the ranking of potential motifs and a search strategy for parameterisation of the motif model. The first two elements can be given an abstract representation, but should probably be designed to utilize and enhance biologically relevant information. The most commonly used motif models are position weight matrices (PWMs) [2,3], mismatch strings (MMs) [4,5] (consensus string allowing some mismatches) and IUPAC strings (IUPACs) [6,7] (consensus string with degenerate symbols).

Due to the large number of available tools, robust assessment of motif discovery methods becomes important, not only for validation of existing tools, but also for pointing out the most promising directions for future research in the field. A major difficulty is our limited knowledge about the biological mechanisms of gene regulation at a detailed level. Although collections of experimentally determined transcription factor binding sites (TFBS) are available, these collections do have inaccuracies and biases. This has been shown e.g. by Fogel *et al*. in their analysis of the TRANSFAC database [8], and by Bergman *et al*. in their study of *Drosophila *gene regulation [9].

A recent article by Tompa *et al*. [10] used experimental collections of TFBS to benchmark a large number of motif discovery tools. This was an important and timely contribution to the field, and it gave good guidance to biologists regarding the level of performance that can be expected with current tools. However, it gave less guidance to the motif discovery field itself. That is, although the study clearly showed a lack of correspondence between *in silico *predictions and *in vivo *experiments, the authors were not able to give much guidance with respect to how we can identify the most promising motif discovery approaches. Furthermore, due to the inherent complexities of the data set, it was hard to distinguish between clever preprocessing and method parameterization done by the expert user on one hand, and the performance of the motif discovery algorithms themselves on the other hand. We note that one of the few clear differences that can be spotted from the generally low performance values – the relatively high score of Weeder – is in the paper partly attributed to judicious choices regarding when to make predictions, while nothing is concluded regarding any superiority of the algorithm itself.

Synthetic data sets may avoid many of these problems. By ensuring that high motif discovery performance is at least theoretically possible, the performance differences between tools may be clearer and more consistent, thus giving more guidance to developers. On the other hand, the coupling may be too loose between the synthetic data sets and the biological reality, introducing an artificial bias. This bias may favor specific classes of tools in a way that lacks biological relevance.

The performance of any motif discovery algorithm can be measured by how well it is able to identify true binding sites in a data set. However, the optimal performance that can be achieved will depend upon the complexity of the data set itself. Here we use a machine learning perspective to analyse collections of TFBS with known binding site locations, in order to estimate an upper bound to the motif discovery performance that can be expected for a given data set. We formulate the problem as a binary classification problem where all sequence windows corresponding to binding sites are termed positive samples, and all other windows are negative samples. Algorithms are given for finding MM, IUPAC and PWM models with optimal discrimination between positive and negative samples.

We use this approach to analyze the experimentally based benchmark data sets used in the recent assessment of motif discovery tools by Tompa *et al*. We also analyze some synthetic benchmark data sets proposed by Pevzner *et al*. [11] and compare the results to those for the experimental collections. Finally we show how the same approach can be used to construct benchmark data sets that combine advantageous properties of both experimentally based and synthetic benchmarks. Data sets are ranked according to the best possible discrimination score as computed by our discrimination approach, and this ranking is used to select subsets with specific properties. We present one benchmark suite with data sets that allow good discrimination between positive and negative instances. This suite, the algorithm benchmark, is useful for evaluating algorithms for motif discovery that rely on the common motif models, as we know that it should be possible to achieve good discrimination with these models. We present another benchmark suite for evaluating motif models, the model benchmark. The data sets in this suite are selected so that none of the common motif models are able to discriminate between positive and negative instances in a reliable way. This suite is useful for evaluating novel and more expressive motif models, as we know that it is not possible to achieve good discrimination with the standard models.

## Results and discussion

We have used the discrimination algorithms described in Methods to analyze motif occurrences in both experimentally based and synthetic benchmark data sets. We present an alternative way of constructing benchmark data sets that uses the discrimination algorithms as a key component.

### Discrimination algorithms

We view a collection of binding sites in a machine learning perspective, where the goal is to find motifs that achieve optimal discrimination of binding sites (at known positions) from remaining sequence. Binding sites are assumed to be of equal length, which may require some alignment and truncation of related sites. Sequence windows corresponding to binding sites are considered positive samples, and all other sequence windows are considered negative samples. For each of the three common motif models, MM, IUPAC and PWM, algorithms have been developed that find the motif that best discriminates between the known positive and negative samples. Discrimination is here defined as finding the single motif that best separates true from false sites, and the discrimination score is the nucleotide-level correlation coefficient (nCC) for this separation, using Formula 1 according to Tompa *et al*. [10]. Details on the problem definition and the individual algorithms are given in Methods and in supplementary material (see additional file 1: IUPAC_details.pdf).

### Analysis of existing benchmark data

We used our discrimination approach to analyze the benchmark suite of Tompa *et al*. For each data set we computed the best possible discrimination between binding sites and remaining sequence using the three motif models. As the binding sites are unaligned and of different length within each individual data set, we had to align and possibly truncate each set of binding sites as a pre-processing step using a gapless alignment [12]. The resulting set of consensus-aligned, equal-length binding site fragments is representative of what can be discovered by standard motif discovery methods.

**Figure 1:**
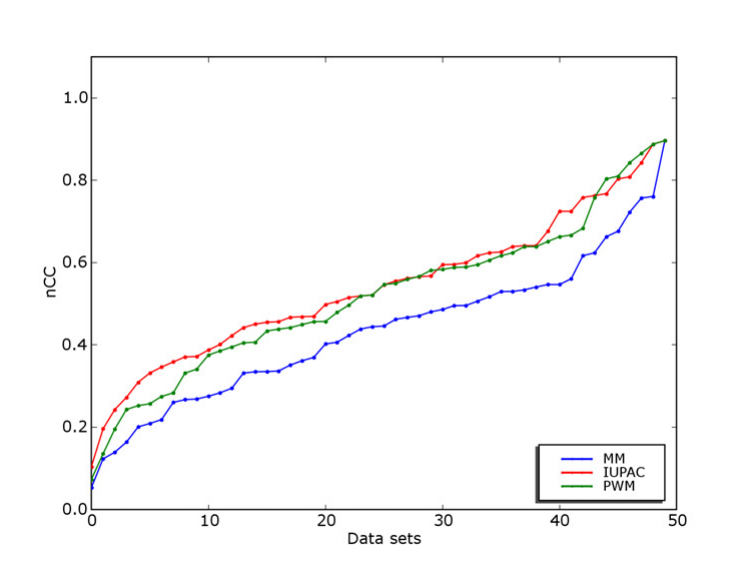
**Discrimination on data sets by Tompa et al**. Nucleotide-level CC-score for discrimination between binding sites and remaining sequence on data sets from Tompa *et al*. Data sets (x-axis) are sorted individually for each model in order of increasing nCC, making it easier to compare the overall distributions of discrimination scores.

**Figure 2:**
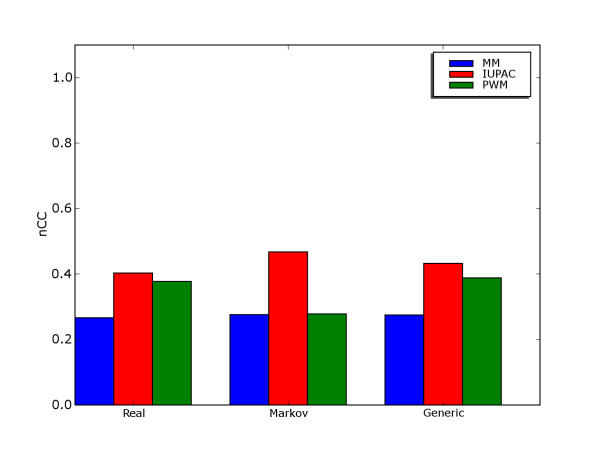
**Discrimination on different data set versions by Tompa et al**. Nucleotide-level CC-score for discrimination between binding sites and remaining sequence for the three motif models on real, generic and Markov versions of data sets.

Figure 1 shows to what extent it is possible to discriminate the set of binding sites from remaining sequence in each of the 50 data sets with a given motif model. We see that this varies a lot, some data sets allow a discrimination score (nCC) of more than 0.8, while other data sets do not allow discrimination score above 0.2 with any of the models. These results are from the "real" data sets from Tompa *et al*. (actual promoter regions), but the scores were similar in the "generic" (binding sites implanted in randomly selected promoter regions) and "Markov" (binding sites implanted in Markov model backgrounds) data sets (see Figure 2).

The IUPAC model had the highest average score, followed by PWM and MM. The score differences between models were statistically significant using paired t-test with 95% confidence level. However, the difference between IUPAC and PWM was very small, and probably not of practical relevance. On the other hand, the score for MM was considerably lower than the others.

Although PWMs are more expressive than IUPAC models, IUPAC scored slightly higher in our tests. PWMs were restricted to either contain log-likelihoods based on aligned binding sites, or to contain log-odds values taking negative data into consideration through a Markov model. All established PWM-based methods use log-likelihood or log-odds matrices, we therefore see this restriction as a reasonable choice. We tried different pseudo-count values and backgrounds with different Markov order, and chose the values that gave best overall score. On the other hand, the algorithms for the IUPAC and mismatch models take negative data directly into consideration, and this leads to slightly better classification performance under certain conditions.

Although the discrimination algorithms return optimal discrimination results on the data they are given, the initial alignment of binding sites in our pre-processing step may be sub-optimal. Multiple alignment algorithms are heuristic, and cannot guarantee optimal solutions. Also, the criteria for optimality of an alignment may not ensure a motif representation that is optimal for classification. As the benchmarked motif discovery methods do not depend on this initial alignment, they may in some cases achieve a somewhat higher nCC-score than what we estimate in the discrimination case (if they can find a better alignment). However, from our experience this is a relatively rare situation, and heuristic ungapped alignment was in general found to perform well on the data sets analyzed here.

#### Cross-validation performance

**Figure 3:**
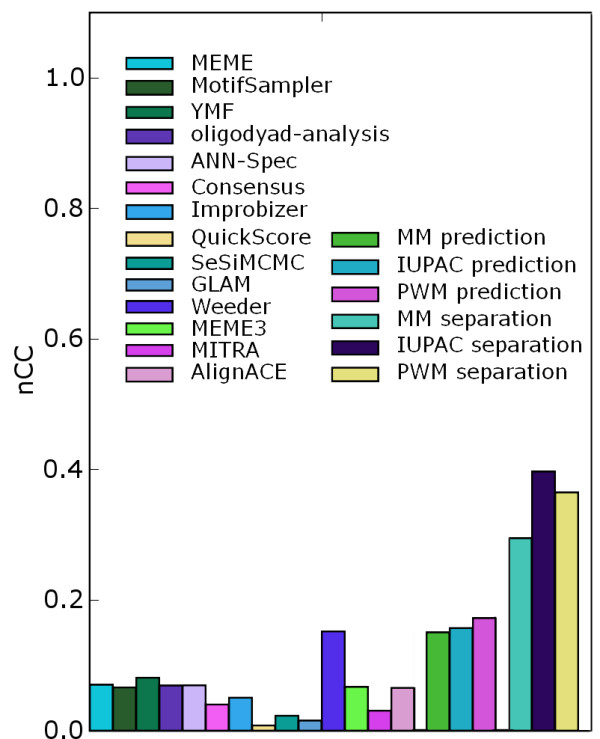
**Motif discovery scores from Tompa et al**. nCC-scores of 14 motif discovery methods given in the Tompa assesment, compared to prediction and discrimination scores with the three main motif models.

Averaged prediction scores for the three motif models in a leave-one-out cross-validation experiment on the benchmark data sets of Tompa *et al*. is given together with discrimination and motif discovery scores in Figure 3. We counted the sum of TP, TN, FP and FN for the test sets across all folds, and calculated the nCC from these accumulated numbers.

As expected, for all models the scores are much lower for cross-validation based prediction than for discrimination. With nCC-scores below 0.2, it shows that even when most binding sites for a TF are known, it is still difficult to predict the location of unseen related binding sites (i.e. it is difficult to generalize from training set to independent test set). Using some strategy to avoid overfitting, e.g. adding regularization terms, could improve the prediction performance somewhat. Still, this means that even if better objective functions [13] could bridge the gap between unsupervised and supervised motif discovery, it would only amount to a limited increase in prediction accuracy on the Tompa benchmark suite. Representation of the sequence similarity between related binding sites seems to be a strong limiting factor. We also see that the IUPAC scores are lower than PWM scores in the cross-validation, confirming that the high IUPAC scores for the discrimination case were partly due to overfitting. Still, the difference in prediction performance between the motif models is very low. Our results thus indicate that the choice of motif model should not be a major limiting factor on motif discovery performance on the benchmark suite of Tompa *et al*. This fits well with the observation that Weeder, which internally uses the simple mismatch model during motif discovery, is able to outperform the many PWM-based methods on this benchmark.

#### Comparison of motif discovery methods

**Figure 4:**
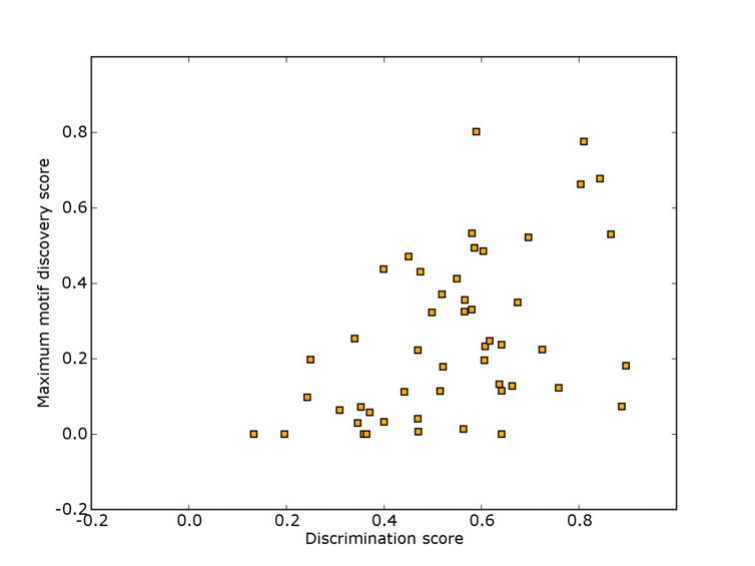
**Motif discovery versus discrimination**. Scatter plot of maximum motif discovery score versus discrimination score for the 50 data sets in the suite by Tompa *et al*.

Figure 3 also shows the scores of different *de novo *motif discovery methods on the benchmark suite of Tompa *et al*., in addition to the average discrimination and prediction scores for each of the three motif models. Although the limited possibility for discrimination between binding sites and remaining sequence puts an upper bound on motif discovery performance on the data sets, the bound is still clearly above the actual scores of these *de novo *motif discovery methods. The discrimination score suggests that motif discovery could be particularly difficult on many of these data sets. We therefore looked at how the maximum score across all motif discovery methods reported in Tompa *et al*. correlated with the discrimination scores on the different data sets. The scatter plot in figure 4 shows that the discrimination score generally represents an upper bound on the motif discovery score, with maximum motif discovery score for data sets typically distributed between zero and the bound given by discrimination score. Only in rare cases may the bound be exceeded due to suboptimal alignments, as already discussed. Most of the motif discovery score values are well below the estimated discrimination score, even though the motif discovery scores we are looking at are maximums over the 14 methods considered in Tompa *et al*.

**Figure 5:**
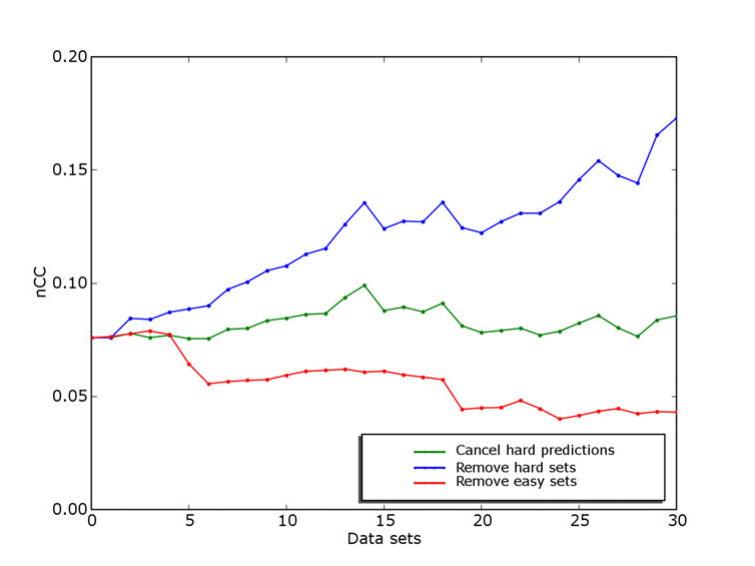
**MEME scores after removals or erasures**. Total MEME score if the data sets with highest or lowest discrimination scores, respectively, had been incrementally removed from the Tompa benchmark, as well as total MEME score if predictions on the data sets with lowest discrimination scores had been incrementally erased.

We also looked at how the total score of a typical motif discovery method would change if data sets were removed according to the discrimination score (Figure 5). We used MEME as example, as it is a well-known method with reasonable performance in the assessment by Tompa *et al*. If only the 13 data sets with lowest discrimination score had been included in the benchmark suite, the nCC-score for MEME would have been just 0.004, compared to a nCC-score of 0.33 if only the 6 data sets with highest discrimination score were used. The nCC-score for MEME on the full benchmark suite was 0.07. We also wanted to explore the remark by Tompa *et al*. that one reason for the good performance of Weeder in the assessment was that the Weeder group was conservative about making predictions. The possible level of discrimination is of course only one of several factors that could influence such a decision, but we wanted to see whether canceling predictions based on discrimination scores alone could have increased the score of MEME on this benchmark suite. We found that the total score of MEME could indeed have been increased slightly by not making any predictions on the data sets with low discrimination score. If no predictions were made on the 14 data sets with lowest discrimination scores, the nCC-score of MEME on the full benchmark suite would have increased by 30%, from 0.076 to 0.099. Actually, because of the generally low performance, MEME would have gotten higher total scores in the assessment (when judged by nCC-score) even if they had submitted blank predictions on all but the 6 data sets with highest discrimination scores.

### Analysis of synthetic benchmark data

Synthetic benchmark data sets avoid many of the problems associated with binding site collections, as the precise locations of synthetic binding sites are known and consistent with the location of sequence consensus. Furthermore, the level of discrimination that is possible to achieve with a given motif model can be controlled.

The problem with synthetic benchmark data is that the generation of synthetic binding sites must necessarily presuppose a model of sequence variability between related sites, for example in the way instances of a base consensus sequence are "mutated" before being implanted in the benchmark sequences. As different motif discovery methods rely on different models of sequence conservation, this will incur a bias towards methods using models similar to the one used when generating data sets. Synthetic benchmark data sets may therefore be suitable for comparing motif discovery methods using the same motif model, but will not give a fair comparison between methods using different motif models.

**Figure 6:**
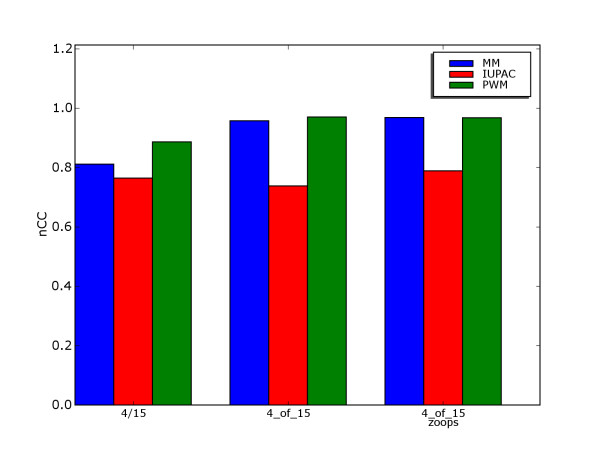
**Discrimination on synthetic data sets**. Discrimination nCC-scores for motif models on three variants of synthetic data sets: variable mutations (4/15), fixed mutations (4_of_15) and fixed mutations with instances in 75% of the sequences (4_of_15 zoops). For each variant, the scores of each model are averaged over 10 randomly generated data sets.

Pevnzer and Sze [11] proposed the Challenge Problem for motif discovery. A data set is constructed by implanting one motif instance in each of 20 sequences, 600 bp long. In the (15,4)-FM version (fixed number of mutations), each motif instance is made by mutating 4 random positions of a 15 bp motif consensus. In the (15,4)-VM version (variable number of mutations), each position of the motif consensus is mutated with a probability of 4/15 when forming a motif instance. Both versions assume that all positions are equally likely to be mutated, and that every nucleotide is equally likely to be the result of a mutation. These are the same assumptions as in the mismatch model. A slight variation to the Challenge Problem is proposed in Styczynski *et al*. [14], where experiments are done on data sets with motif instances in only 15 out of 20 sequences. Figure 6 shows the discrimination scores of the three common motif models, averaged over 10 data sets of 20 sequences randomly constructed according to the three variants of the Challenge Problem. Contrary to the results on annotated binding site collections, the MM model gets very competitive discrimination scores on the Challenge Problem data sets, only slightly lower than PWM scores. The IUPAC model, which had the highest average discrimination score on the data sets from Tompa *et al*., gets the lowest score on the synthetic data sets. The IUPAC model is the model that most clearly relies on asymmetries in positional conservation and skewed positional nucleotide distributions, properties not present in these synthetic data sets, although they are assumed to be biologically relevant. Both the high empirical scores of the mismatch model, and the low scores of the IUPAC model, support the intuition that synthetic data sets may introduce a bias towards a presupposed model.

### Generation of improved benchmark data

**Figure 7:**
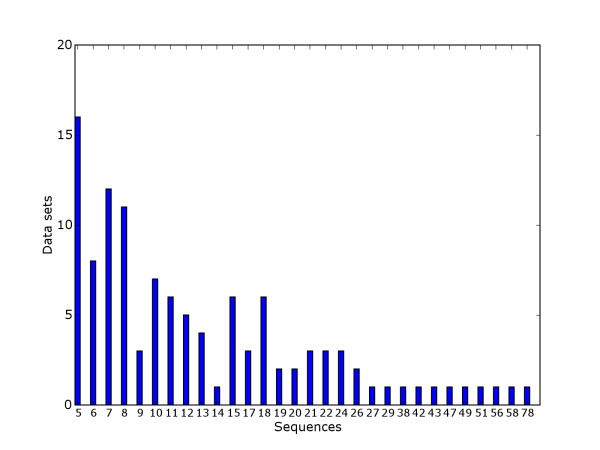
**Sequences per data set**. Distribution of number of sequences per data set.

**Figure 8:**
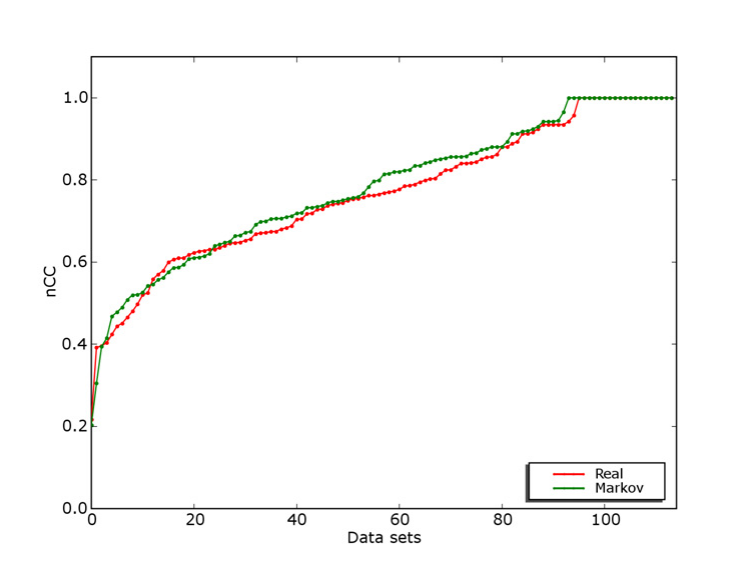
**Discrimination on all TRANSFAC-based data sets**. Nucleotide-level CC-score for discrimination between binding sites and remaining sequence on real and Markov version of TRANSFAC-based data sets. For each data set, the highest discrimination score achieved by any of the three motif models is selected. The distribution of scores are in sorted order for real and Markov versions independently.

Based on our analysis of existing benchmark data we propose a new strategy for the generation of benchmark suites. Details are given in Methods. Basically binding site fragments corresponding to known binding sites were extracted from a suitable database (TRANSFAC) and represented either as real sequences (i.e. binding sites in their original genomic context) or Markov sequences (binding sites implanted in sequences generated with a third order Markov model). Figure 7 shows the distribution of binding sites. The best sequence-based discrimination between binding sites and remaining sequence was computed, as shown in Figure 8. Based on the discrimination score two subsets were generated, an algorithm benchmark suite and a model benchmark suite.

#### The algorithm benchmark suite

**Figure 9:**
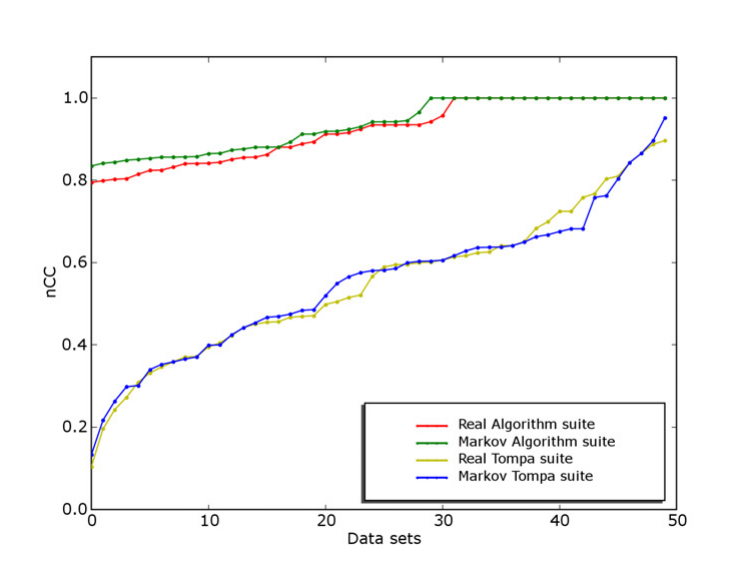
**Discrimination on algorithm benchmark suite**. Nucleotide-level CC-score for discrimination between binding sites and remaining sequence. Results are given for our algorithm benchmark suite and the suite by Tompa *et al*., for both real and Markov versions. For each data set, the highest discrimination score achieved by any of the three motif models is selected. The distribution of scores are in sorted order for all versions independently.

For our algorithm benchmark suite we selected all data sets with discrimination score higher than 0.79 for the real version and higher than 0.87 for the Markov version, giving 50 data sets of each version. Figure 9 compares the distribution of discrimination scores for this suite to the suite by Tompa *et al*., showing that the binding sites are standing out from background much more clearly in our algorithm benchmark suite.

This gives a benchmark suite where we know that it is possible to achieve good discrimination with standard motif models. This suite will therefore mainly evaluate the performance of the algorithms for motif discovery, as lack of performance has to be caused by failure to find optimal motifs, and not the motif model itself.

#### The model benchmark suite

The field would also gain from more powerful motif models that can better capture the variability between binding sites and discriminate these from background. This will be even more relevant as more examples of related binding sites become available.

**Figure 10:**
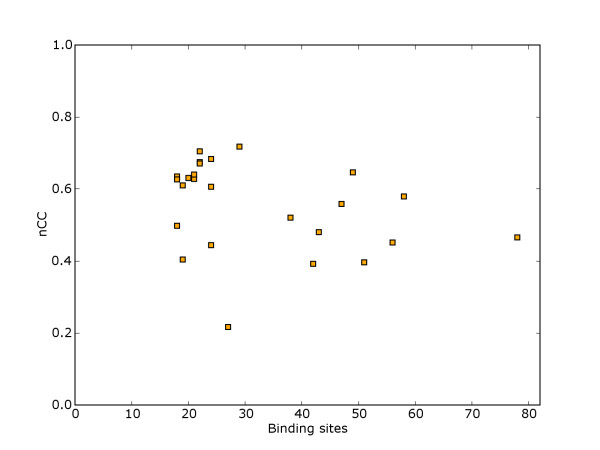
**Discrimination score and number of binding sites**. Distribution of discrimination scores (nCC) and number of binding sites for each of the 25 data sets in the model benchmark suite.

For benchmarking of novel powerful motif models, we propose a model benchmark suite with binding sites that are hard to discriminate from background. The construction was similar to the preceding suite, except that for this suite data sets were selected that only allow a low level of discrimination with the common motif models. As powerful models typically require the estimation of more parameters, we also filtered out data sets with few binding sites. We selected 25 data sets with at least 18 binding sites in each data set, and with discrimination score below 0.72 for the three common motif models. Figure 10 shows the distribution of the number of binding sites and the maximum discrimination score with common models for each data set in the model benchmark suite. Table 1 shows the aggregated results in comparison to algorithm benchmark suites. As more experimentally determined binding sites become available in the future, the same methodology could give benchmark suites with a larger number of binding sites per data set, and even lower maximum discrimination scores when using the common models.

**Table 1:** Discrimination scores on model benchmark suite. Average nCC-scores of three motif models on our proposed model benchmark suites; real and Markov algorithm suite, as well as real model suite.

	Algorithm suite (Real)	Algorithm suite (Markov)	Model suite (Real)
PWMs	0.89	0.90	0.48
IUPACs	0.87	0.87	0.50
MMs	0.67	0.64	0.33

**Figure 11:**
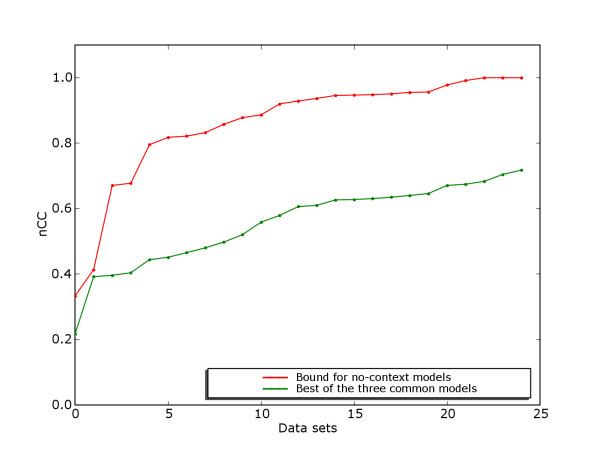
**Discrimination score on model benchmark suite**. Distribution of discrimination scores (nCC) for the 25 data sets in the model benchmark suite. One curve shows the best score of the three common motif models on the data set, while the other curve shows the score possible with a more expressive model that still do not consider the context of binding sites.

For several data sets, some of the substrings marked as binding site also had an exact unannotated duplicate in another sequence. This means that without working with longer motif length, or operating with a motif context based on flanking sequence, it is not possible to achieve perfect discrimination with any model. The distribution of maximum discrimination scores possible with any model without taking such measures, as well as the maximum discrimination possible with the currently common motif models, is given in Figure 11.

### Examples of benchmark runs

We ran MEME and Weeder on our proposed benchmark suite to indicate the level of motif discovery performance that can be expected. Table 2 compares the scores of MEME and Weeder with the discrimination scores of the PWM model. As expected, the *de novo *motif discovery scores are much lower than the upper bound given by the discrimination score. Note that all motif discovery results given on our benchmark suites have been achieved with default parameters. Slightly higher scores might be achieved by tweaking of parameters and clever post-processing of results.

**Table 2:** Discrimination and motif discovery scores on algorithm benchmark suite. Average nCC-scores for *de novo *motif discovery with MEME and Weeder compared to best discrimination scores on our proposed algorithm benchmarks and remaining 64 datasets.

	Algorithm suite (Real)	Remaining data sets (Real)	Algorithm suite (Markov)
MEME	0.068	0.029	0.082
Weeder	0.11	0.10	0.052
Disc.	0.92	0.64	0.92

The average score of MEME is higher on the real Algorithm suite than on the remaining real data sets. For Weeder this difference was less clear. While MEME achieves slightly higher scores on Markov version compared to real version of Algorithm suite, Weeder performs better on the real version. This might possibly be reflecting the different approaches to estimation of background distribution in MEME and Weeder.

Although the performance of both MEME and Weeder is better than random even with default parameters on real sequences, the performance is still much lower than the bounds given by the discrimination scores, leaving much room for improvement in the development of objective functions and search heuristics for motif discovery.

## Conclusion

We have developed discrimination algorithms for the common motif models and used these algorithms both for analyzing an existing benchmark suite and for constructing new benchmark suites. The work has highlighted several important points:

• Considering discrimination of known binding sites from background separates the limitations of motif models from the limitations of objective functions and search heuristics. Discrimination algorithms for common motif models may be used to evaluate properties of data sets, for instance in a filtering step when constructing benchmark data sets.

• Motif discovery is very difficult on the data sets used in the recent benchmark of Tompa *et al*. Algorithms reveal large difficulties even with the basic task of discriminating a set of known binding sites from remaining sequence.

• Improved benchmark data sets with controlled properties can be constructed from motif databases, e.g. TRANSFAC matrix alignments, using discrimination algorithms for filtering. Using this approach, we propose one benchmark suite for evaluating the motif discovery process itself with current models, and another benchmark suite with data sets that could profit from more expressive motif models.

Our main focus has been on the level of discrimination that is possible for a given data set, and we have used the maximum score across the three models to avoid introducing a bias towards a specific model during the evaluation and filtering of benchmark data sets. Still, we have observed some consistent differences between the discrimination power of the common models: The IUPAC model achieves the highest level of discrimination, slightly above the PWM model, with the mismatch model at a clearly lower level. On the other hand, synthetic benchmark data sets rely on a chosen computational method for generating variability among implanted binding sites. As expected, the motif models that are more compatible with the generation model achieved better discrimination scores on three versions of synthetic data sets that were considered.

A main line of future work would be to increase the size and quality of benchmark data sets by using our proposed methodology on additional binding site collections. Also, as time goes, more data of higher quality will be available in the TRANSFAC database used in this work as well as in other similar databases. A different line of research would be to use a supervised learning approach as a first step in exploring novel and more expressive motif models. After the power of a new motif model has been determined by its discrimination scores on training sets, and its generalization ability has been determined by its prediction scores on independent test sets, the more complex task of developing efficent methods for *de novo *discovery could be commenced. Supervised learning algorithms could be developed for entirely new models, or for exploring already proposed expressive models such as HMDM [15], Bayesian nets [16,17], Markov-model motifs [18,19], dinucleotide matrices [20,21] and SPSP [22].

## Methods

### Motif models

The most common models of motifs in DNA sequences are PWM, IUPAC codes and mismatch strings. These are considered as three different hypothesis spaces in our work. Deciding on the hypothesis space is central to machine learning [23]. A good hypothesis space for a domain should be as small as possible while still containing a good hypothesis. The main motivation in this work is to find the best hypothesis in the respective hypothesis space of motif models. We have developed exhaustive search algorithms to avoid any search bias. Since for large model sizes exploring the whole search space becomes impractical, the algorithms developed are optimized as much as possible so as to scale well for moderate sizes.

#### Problem formulation

**Figure 12:**
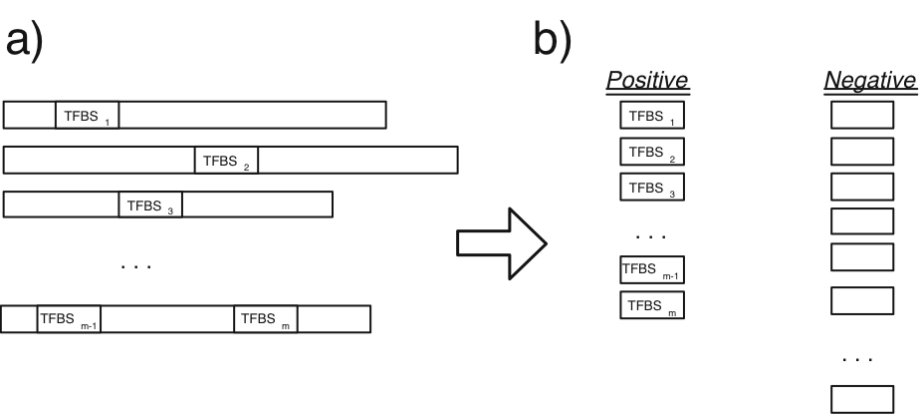
**Generating positive and negative examples**. A set of upstream DNA sequences for a transcription factor where a) *m *binding locations are identified, b) generating positive and negative examples.

We assume that a number of upstream DNA sequences with binding site locations are given. The locations are positive examples while other oligos with the same length in the same sequence set form the negative examples (Figure 12).

Let *E *be a set of *N *TFs, *i.e*., *E *= {*TF*_1_, *TF*_2_,...,*TF*_*N*_}. Associated with each TF is its binding site length *k *: *E *→ ℕ, usually ranging between 6 and 20 and assumed to be known. The input space for *TF*_*i *_is X(TFi)={A,C,G,T}k(TFi)
 MathType@MTEF@5@5@+=feaafiart1ev1aaatCvAUfKttLearuWrP9MDH5MBPbIqV92AaeXatLxBI9gBaebbnrfifHhDYfgasaacH8akY=wiFfYdH8Gipec8Eeeu0xXdbba9frFj0=OqFfea0dXdd9vqai=hGuQ8kuc9pgc9s8qqaq=dirpe0xb9q8qiLsFr0=vr0=vr0dc8meaabaqaciaacaGaaeqabaqabeGadaaakeaacqWGybawcqGGOaakcqWGubavcqWGgbGrdaWgaaWcbaGaemyAaKgabeaakiabcMcaPiabg2da9iabcUha7jabdgeabjabcYcaSiabdoeadjabcYcaSiabdEeahjabcYcaSiabdsfaujabc2ha9naaCaaaleqabaGaem4AaSMaeiikaGIaemivaqLaemOray0aaSbaaWqaaiabdMgaPbqabaWccqGGPaqkaaaaaa@458D@, *i *∈ {1, 2,...,*N*}. The output space *Y *= {0, 1}, indicating negative/positive examples.

For *TF*_*i*_, let the learner be a function ATFi:{A,C,G,T}k(TFi)
 MathType@MTEF@5@5@+=feaafiart1ev1aaatCvAUfKttLearuWrP9MDH5MBPbIqV92AaeXatLxBI9gBaebbnrfifHhDYfgasaacH8akY=wiFfYdH8Gipec8Eeeu0xXdbba9frFj0=OqFfea0dXdd9vqai=hGuQ8kuc9pgc9s8qqaq=dirpe0xb9q8qiLsFr0=vr0=vr0dc8meaabaqaciaacaGaaeqabaqabeGadaaakeaacqWGbbqqdaWgaaWcbaGaemivaqLaemOray0aaSbaaWqaaiabdMgaPbqabaaaleqaaOGaeiOoaOJaei4EaSNaemyqaeKaeiilaWIaem4qamKaeiilaWIaem4raCKaeiilaWIaemivaqLaeiyFa03aaWbaaSqabeaacqWGRbWAcqGGOaakcqWGubavcqWGgbGrdaWgaaadbaGaemyAaKgabeaaliabcMcaPaaaaaa@43DB@ → ℋ
 MathType@MTEF@5@5@+=feaafiart1ev1aaatCvAUfKttLearuWrP9MDH5MBPbIqV92AaeXatLxBI9gBamrtHrhAL1wy0L2yHvtyaeHbnfgDOvwBHrxAJfwnaebbnrfifHhDYfgasaacH8akY=wiFfYdH8Gipec8Eeeu0xXdbba9frFj0=OqFfea0dXdd9vqai=hGuQ8kuc9pgc9s8qqaq=dirpe0xb9q8qiLsFr0=vr0=vr0dc8meaabaqaciaacaGaaeqabaWaaeGaeaaakeaaimaacqWFlecsaaa@3763@, for a predefined hypothesis space ℋ
 MathType@MTEF@5@5@+=feaafiart1ev1aaatCvAUfKttLearuWrP9MDH5MBPbIqV92AaeXatLxBI9gBamrtHrhAL1wy0L2yHvtyaeHbnfgDOvwBHrxAJfwnaebbnrfifHhDYfgasaacH8akY=wiFfYdH8Gipec8Eeeu0xXdbba9frFj0=OqFfea0dXdd9vqai=hGuQ8kuc9pgc9s8qqaq=dirpe0xb9q8qiLsFr0=vr0=vr0dc8meaabaqaciaacaGaaeqabaWaaeGaeaaakeaaimaacqWFlecsaaa@3763@.

We restrict our hypothesis space set to ℍ = {ℋ
 MathType@MTEF@5@5@+=feaafiart1ev1aaatCvAUfKttLearuWrP9MDH5MBPbIqV92AaeXatLxBI9gBamrtHrhAL1wy0L2yHvtyaeHbnfgDOvwBHrxAJfwnaebbnrfifHhDYfgasaacH8akY=wiFfYdH8Gipec8Eeeu0xXdbba9frFj0=OqFfea0dXdd9vqai=hGuQ8kuc9pgc9s8qqaq=dirpe0xb9q8qiLsFr0=vr0=vr0dc8meaabaqaciaacaGaaeqabaWaaeGaeaaakeaaimaacqWFlecsaaa@3763@_*PWM*_, ℋ
 MathType@MTEF@5@5@+=feaafiart1ev1aaatCvAUfKttLearuWrP9MDH5MBPbIqV92AaeXatLxBI9gBamrtHrhAL1wy0L2yHvtyaeHbnfgDOvwBHrxAJfwnaebbnrfifHhDYfgasaacH8akY=wiFfYdH8Gipec8Eeeu0xXdbba9frFj0=OqFfea0dXdd9vqai=hGuQ8kuc9pgc9s8qqaq=dirpe0xb9q8qiLsFr0=vr0=vr0dc8meaabaqaciaacaGaaeqabaWaaeGaeaaakeaaimaacqWFlecsaaa@3763@_*IUPAC*_, ℋ
 MathType@MTEF@5@5@+=feaafiart1ev1aaatCvAUfKttLearuWrP9MDH5MBPbIqV92AaeXatLxBI9gBamrtHrhAL1wy0L2yHvtyaeHbnfgDOvwBHrxAJfwnaebbnrfifHhDYfgasaacH8akY=wiFfYdH8Gipec8Eeeu0xXdbba9frFj0=OqFfea0dXdd9vqai=hGuQ8kuc9pgc9s8qqaq=dirpe0xb9q8qiLsFr0=vr0=vr0dc8meaabaqaciaacaGaaeqabaWaaeGaeaaakeaaimaacqWFlecsaaa@3763@_*MM*_} representing PWM, IUPAC and mismatch string models.

We use correlation coefficient (CC) as our performance metric. In the optimization, CC is calculated at the oligo (sequence window) level, using oligos as individual samples as explained previously in this section. This differs slightly from the CC at the nucleotide level (nCC), which is the measure used in the results section to ensure consistency with the results of Tompa *et al*.

nCC=TP⋅TN−FP⋅FN(TP+FP)⋅(FP+TN)⋅(TN+FN)⋅(FN+TP)
 MathType@MTEF@5@5@+=feaafiart1ev1aaatCvAUfKttLearuWrP9MDH5MBPbIqV92AaeXatLxBI9gBaebbnrfifHhDYfgasaacH8akY=wiFfYdH8Gipec8Eeeu0xXdbba9frFj0=OqFfea0dXdd9vqai=hGuQ8kuc9pgc9s8qqaq=dirpe0xb9q8qiLsFr0=vr0=vr0dc8meaabaqaciaacaGaaeqabaqabeGadaaakeaacqWGUbGBcqWGdbWqcqWGdbWqcqGH9aqpdaWcaaqaaiabdsfaujabdcfaqjabgwSixlabdsfaujabd6eaojabgkHiTiabdAeagjabdcfaqjabgwSixlabdAeagjabd6eaobqaamaakaaabaGaeiikaGIaemivaqLaemiuaaLaey4kaSIaemOrayKaemiuaaLaeiykaKIaeyyXICTaeiikaGIaemOrayKaemiuaaLaey4kaSIaemivaqLaemOta4KaeiykaKIaeyyXICTaeiikaGIaemivaqLaemOta4Kaey4kaSIaemOrayKaemOta4KaeiykaKIaeyyXICTaeiikaGIaemOrayKaemOta4Kaey4kaSIaemivaqLaemiuaaLaeiykaKcaleqaaaaaaaa@6387@

### Algorithms

#### Mismatch string model

The motif model for mismatch strings is a tuple, *M*_*MM *_= <*cs*, *d *> where *cs *∈ {*A, C, G, T*}^*n *^is a consensus string of length *n *and *d *∈ {0, 1,..., *n*} is the maximum Hamming distance from *cs*. Typical values for *n *is 6 to 20 and that of *d *is 1 to 4. Bounded values for *n *and *d *clearly suggests that hypothesis space is finite, although large when *n *gets bigger.

For mismatch strings we have developed an algorithm inspired by [24]. A main difference is that in our case, the motif locations are assumed known, *i.e*., a supervised case. The main idea is to enumerate every substring *s *within a given Hamming distance *d *of each positive substring in the data set. For each such substring *s*, matches are determined as every substring *s' *of the sequences at a Hamming distance of at most *d *from *s*.

The method described above clearly does not consider all the hypothesis space explicitly, but the subset considered is actually enough to find the best hypothesis among all. Since the best hypothesis must cover at least one positive instance, the algorithm is guaranteed to find the best hypothesis even though not all hypotheses are explicitly enumerated. Thus, it suffices to evaluate the score of this subset of hypotheses. As this still involves scanning very many different motifs against the same sequences, a *q-gram *of the sequences is used to further accelerate matching of short motifs (length < 7) against sequences, and the algorithm of Yates *et al*. [25] for longer motifs.

#### IUPAC model

The motif model for IUPAC, *M*_*IUPAC*_, is a degenerate string *ds *of length *n *where each position is a non-empty subset of {*A, C, G, T*}. These subsets correspond to the IUPAC symbols for DNA sequences. For finite *n*, the hypothesis space is finite but grows exponentially with *n*. A candidate string *s *is said to be a hit (match) against *ds *if every position of *s *is a subset of respective position in *ds*, otherwise it is a non-hit (non-match).

Finding a IUPAC expression that perfectly separates positive and negative substrings of equal length is indeed straightforward if at all possible for a data set. IUPAC expressions are a subset of regular expressions, and induction of regular expressions from sequence examples is well studied. Each position of the motif is set to the union of characters occurring at the respective position of the positive instances. To see that this is the only solution, note that leaving out any symbol that occurs in a positive instance at a given position leads to the motif not covering the instance. Additionally, adding symbols not occurring in any positive instance at that position may only introduce hits in negative instances.

Perfect classification is generally impossible for the problem we consider. In our case, all degenerate strings should be generated exhaustively and evaluated against a scoring function. We have therefore developed an efficient algorithm that avoids unnecessary exploration of the hypothesis space. Our algorithm bears some similarities with the SPEXS algorithm for *de novo *motif discovery [26], but differs in that it uses bit-strings and pre-computation for optimization, calculates bounds and prunes subtrees, and of course that it solves a classification problem with known positive and negative instances instead of an unsupervised data mining problem. The details of the algorithm can be found in the supplementary material.

#### PWM model

The motif model for PWM is a tuple *M*_*PWM *_= <*M, t *> where *M *is a matrix of 4 × *n *where each column is a probability distribution of the nucleotide vector <*A, C, G, T *> and *n *is the length of the motif. A candidate string is considered to be a hit if the sum of probabilities in respective rows are greater than the threshold *t*, otherwise a non-hit. The hypothesis space is infinite regardless of *n*.

PWMs used for motif discovery are not just arbitrary matrices that best separates the motif occurrences from the remaining sequences. On the contrary, a PWM has a clear interpretation as a product multinomial probability distribution, or as containing log-odds values of motif versus background. In the supervised case we calculate the PWM from symbol frequencies in known motif locations for log-probability matrix. Additionally, background distributions are taken into account for log-odds PWM matrix. As the PWM thus is a direct function of the positive (and negative) instances of the data set, it is calculated easily and efficiently even for large data sets. We used the highest scoring PWM version for discrimination score. In motif discovery, the hypothesized motif locations used for constructing a PWM can in general be any probability distribution over all sequence locations. If the hypothesized motif locations exactly match the annotated sites, it corresponds to the solution in the supervised case.

Although the PWM itself is calculated directly from sequence data, there is more flexibility when it comes to determining a PWM score threshold to be used when determining binary hits of the PWM. Such score thresholds are commonly used to get a list of motif locations, instead of just a distribution on motif locations across the whole sequence data. As there are many ways of determining score thresholds, we exhaustively find the threshold that optimize the score of a given PWM.

We do this by exploiting the fact that the optimal threshold must be equal to the PWM match score of a positive instance. We therefore compute the classification score of the PWM with each of these thresholds and choose the threshold giving highest classification score. To see why this is optimal, consider a threshold *t *that is not equal to the PWM score of any positive instance. Increasing this threshold to the PWM score of the positive instance with least margin to the threshold (*t'*) will give the same number of TP. As the threshold is more stringent the number of TN must be equal or higher. Thus, there exists a threshold *t'*, corresponding to the PWM score of a positive instance, with at least as high score as the threshold *t*.

### Dataset generation

We extracted sets of binding site fragments for 213 different TF matrices from the TRANSFAC database, version 9.4 [27]. A binding site fragment is the binding site region that is used in the construction of a matrix in the TRANSFAC alignment. Both real and Markov data set versions were constructed from the same fragment sets. For the real version, binding sites were kept in their original genomic sequence, which was truncated to a maximum length of 2000 bp. To make the data sets more coherent, we removed binding site fragments that contained degenerate bases, that had gaps in the TRANSFAC alignment, that were not located within the 2000 bp upstream of transcription start site in the sequence linked to by TRANSFAC, or that had two or more occurrences in the 2000 bp region. The binding sites used in a TRANSFAC matrix alignment may occur on opposite strands. To simplify the process of using these data sets we took the reverse complement of linked sequences when the binding site appeared on the negative strand. For the Markov version, binding sites were implanted in sequences generated from a third order Markov model inferred from all sequences of the corresponding real data set. Both the lengths of the Markov version sequences and the positions of the implanted binding sites were kept equal to the corresponding real sequences. Data sets with fewer than five binding sites were removed, leaving us with 114 real and 114 Markov data sets. While most data sets had from 5 to 25 sequences, there were data sets with up to 78 sequences. We then computed the best possible discrimination score, and used that for selecting the algorithm and model suites, as described in the main text.

### Parameter settings

For all our runs of MEME we used version 3.5.3, downloaded from [28]. To avoid incurring biases in our results, we ran MEME with default DNA parameter values and without any manual curation of output data. We think this is realistic with regard to common usage of motif discovery methods, although performance could probably have been improved by tweaking parameters, pre-processing data sets and post-processing output data. For all runs of Weeder we used version 1.3 downloaded from [29]. We also ran Weeder with default parameters and without any manual curation. We used the large setting and the option telling Weeder that each sequence should contain at least one binding site. As Weeder requires the specification of organism, we supplied for each data set the most frequent organism.

## Availability and requirements

Our proposed algorithm and model benchmark suites are available for download at . We have also implemented a web service for evaluating predictions and visualizing benchmark results. The implementation of the discrimination algorithms for the common motif models is freely available as Python source code at the same address.

## Abbreviations

PWM: position weight matrix;

IUPAC: nomenclature for degenerate symbols as defined by the International Union of Pure and Applied Chemistry;

MM: mismatch motif model;

TFBS: transcription factor binding site;

TP, TN, FP, FN: true/false positives/negatives;

nCC: nucleotide-level Pearson's correlation coefficient (Formula 1)

## Authors' contributions

GKS conceived the initial idea together with FD, devised the discrimination algorithms and drafted the manuscript. OA contributed to the scientific content of the paper, formalized the machine learning perspective and took part in writing the manuscript. VW revised and implemented the algorithms. FD supervised and took part in all stages of the project.

## Supplementary Material

Additional file 1 **Details on discrimination algorithm for IUPAC model**. The file IUPAC_details.pdf describes the discrimination algorithm for IUPAC model in more detail, as well as several optimizations of computational efficiency. Click here for file

## References

[B1] Sandve GK, Drabløs F (2006). A survey of motif discovery methods in an integrated framework. Biol Direct.

[B2] Hughes JD, Estep PW, Tavazoie S, Church GM (2000). Computational identification of cis-regulatory elements associated with groups of functionally related genes in Saccharomyces cerevisiae. J Mol Biol.

[B3] Bailey TL, Elkan C (1995). The value of prior knowledge in discovering motifs with MEME. Proc Int Conf Intell Syst Mol Biol.

[B4] Marsan L, Sagot MF (2000). Extracting structured motifs using a suffix tree-algorithms and application to promoter consensus identification. RECOMB '00: Proceedings of the fourth annual international conference on Computational molecular biology.

[B5] Blanchette M, Tompa M (2002). Discovery of regulatory elements by a computational method for phylogenetic footprinting. Genome Res.

[B6] Sinha S, Tompa M (2003). YMF: A program for discovery of novel transcription factor binding sites by statistical overrepresentation. Nucleic Acids Res.

[B7] Bortoluzzi S, Coppe A, Bisognin A, Pizzi C, Danieli G (2005). A Multistep Bioinformatic Approach Detects Putative Regulatory Elements In Gene Promoters. BMC Bioinformatics.

[B8] Fogel GB, Weekes DG, Varga G, Dow ER, Craven AM, Harlow HB, Su EW, Onyia JE, Su C (2005). A statistical analysis of the TRANSFAC database. Biosystems.

[B9] Bergman CM, Carlson JW, Celniker SE (2005). Drosophila DNase I footprint database: a systematic genome annotation of transcription factor binding sites in the fruitfly, Drosophila melanogaster. Bioinformatics.

[B10] Tompa M, Li N, Bailey TL, Church GM, De Moor B, Eskin E, Favorov AV, Frith MC, Fu Y, Kent WJ, Makeev VJ, Mironov AA, Noble WS, Pavesi G, Pesole G, Regnier M, Simonis N, Sinha S, Thijs G, van Helden J, Vandenbogaert M, Weng Z, Workman C, Ye C, Zhu Z (2005). Assessing computational tools for the discovery of transcription factor binding sites. Nat Biotechnol.

[B11] Pevzner PA, Sze SH (2000). Combinatorial approaches to finding subtle signals in DNA sequences. Proc Int Conf Intell Syst Mol Biol.

[B12] Henikoff S, Henikoff JG, Alford WJ, Pietrokovski S (1995). Automated construction and graphical presentation of protein blocks from unaligned sequences. Gene.

[B13] Li N, Tompa M (2006). Analysis of computational approaches for motif discovery. Algorithms Mol Biol.

[B14] Styczynski MP, Jensen KL, Rigoutsos I, Stephanopoulos GN (2004). An extension and novel solution to the (l,d)-motif challenge problem. Genome Inform.

[B15] Xing EP, Jordan MI, Karp RM, Russell S, Becker S, Thrun S, Obermayer K (2002). A hierarchical bayesian markovian model for motifs in biopolymer sequences. Advances in Neural Information Processing Systems.

[B16] Barash Y, Elidan G, Friedman N, Kaplan T (2003). Modeling dependencies in protein-DNA binding sites. RECOMB '03: Proceedings of the seventh annual international conference on Computational molecular biology.

[B17] Ben-Gal I, Shani A, Gohr A, Grau J, Arviv S, Shmilovici A, Posch S, Grosse I (2005). Identification of transcription factor binding sites with variable-order Bayesian networks. Bioinformatics.

[B18] Lim LP, Burge CB (2001). A computational analysis of sequence features involved in recognition of short introns. Proc Natl Acad Sci USA.

[B19] Zhao X, Huang H, Speed TP (2004). Finding short DNA motifs using permuted markov models. RECOMB '04: Proceedings of the eighth annual international conference on Computational molecular biology.

[B20] Stormo GD, Schneider TD, Gold L (1986). Quantitative analysis of the relationship between nucleotide sequence and functional activity. Nucleic Acids Res.

[B21] Zhou Q, Liu JS (2004). Modeling within-motif dependence for transcription factor binding site predictions. Bioinformatics.

[B22] Leung HC, Chin FY (2006). Discovering DNA Motifs with Nucleotide Dependency. Sixth IEEE Symposium on Bioinformatics and Bioengineering (BIBE), IEEE Computer Society.

[B23] Mitchell TM (1997). Machine Learning.

[B24] Keich U, Pevzner PA (2002). Finding motifs in the twilight zone. Bioinformatics.

[B25] Baeza-Yates RA, Perleberg CH (1992). Fast and Practical Approximate String Matching. CPM '92: Proceedings of the Third Annual Symposium on Combinatorial Pattern Matching.

[B26] Vilo J (1998). Discovering Frequent Patterns from Strings. Tech Rep C-1998-9.

[B27] Wingender E, Dietze P, Karas H, Knuppel R (1996). TRANSFAC: a database on transcription factors and their DNA binding sites. Nucleic Acids Res.

[B28] MEME. http://meme.nbcr.net/downloads/.

[B29] Weeder. http://159.149.109.16:8080/weederWeb/.

