# Supplementary material for: Improved benchmarks for computational motif discovery
Source: BMC Bioinformatics. 2007 Jun 8;8:193. doi: 10.1186/1471-2105-8-193 (PMC1903367; doi:10.1186/1471-2105-8-193)
Supplement: Additional file 1 — Details on discrimination algorithm for IUPAC model. The file IUPAC_details.pdf describes the discrimination algorithm for IUPAC model in more detail, as well as several optimizations of computational efficiency. [file 1471-2105-8-193-S1.pdf]

Details on discrimination algorithm for IUPAC model

Geir Kjetil Sandve, Osman Abul, Vegard Walseng and Finn Drabløs

The search space of IUPAC motifs is explored depth-first in a (implicit) search tree where each level of the tree corresponds to a position in the motif (Figure 1). Each node has 15 children, corresponding to the possible IUPAC symbols at the position corresponding to the next level in the tree. Consequently, a node nd at level i of the tree then corresponds to a motif of length i, with symbols at each positions given by the nodes on the path from the root to node nd.

The computation being done in each node is given in Algorithm 1. First, the score of the node is computed and the best motif is updated (lines 10-14) and returned (line 27). For each non-leaf node, the nodes to branch to are determined and recursed (lines 15-26). The instances in the current positive hit list are projected at position $Level + 1$ and only nucleotides appearing at least once in that position and their combinations are considered (lines 16-17) to reduce branching factor. Each of these IUPAC symbols are evaluated against the bounding condition (line 22) after computing their hit counts in positive (line 19) and negative (line 20) sets. The IUPAC symbols that are not bounded are recursed at the next level (line 23).

The algorithm is initialized with the following inputs; n equal to motif width, $Level = 0$, $Best\_Score$ equal to the smallest possible value for the scoring function (*i.e.* -1 for CC), $Best\_Solution = \emptyset$, $Edge\_Labels = \emptyset$, $Hits\_Pset$ ($Hits\_Nset$) equal to the set of all positive (negative) instances, $Size\_Pset = |Hits\_Pset|$, and $Size\_Nset = |Hits\_Nset|$.

There are mainly 5 optimizations of efficiency we employ in the algorithm, *Bit-strings, Bounding, Projection to next level, Pre-computation*, and *Counting without scanning.*

Bit-strings: The algorithm operates on bit-strings to increase the computational efficiency, *i.e.* $Hits\_Pset$ and $Hits\_Nset$ are represented by bit-strings. A bit-string for a node is calculated by intersecting the bit-string of the parent node and a pre-computed bit-string for the IUPAC symbol of the node at its corresponding position. The count of 1s in the bit-string for positive substrings gives TP, while the count of 1s in the bit-string for negative substrings gives FP.

Bounding: The algorithm itself is a branch and bound algorithm. It prunes any node for which no node in its subtree can improve on the current score. This is achieved by keeping the maximum score (lines 10-14) found so far throughout the search. As a substring that do not match the first positions of a motif can never match the whole motif, the current TP of a node nd is an upper bound for every successor of nd. By a similar argument, a substring

Algorithm 1 Algorithm for efficient identification of optimum IUPAC motif

Traverse_IUPAC(...)

INPUT: n : motif length
INPUT: $Level$: Depth of the current node
INPUT: $Best\_Score$: Best score so far
INPUT: $Best\_Solution$: Best solution so far
INPUT: $Edge\_Labels$: Ordered labels on edges from the root to this node
INPUT: $Hits\_Pset$: Subset of instances in positive set matching all ancestors in respective columns
INPUT: $Hits\_Nset$: Subset of instances in negative set matching all ancestors in respective columns
INPUT: $Size\_Pset$: Number of instances in positive set
INPUT: $Size\_Nset$: Number of instances in negative set
$score \leftarrow Compute\_Score(|Hits\_Pset|, |Hits\_Nset|, Size\_Pset, Size\_Nset)$
if $score > Best\_Score$ **then**
    $Best\_Score \leftarrow score$
    $Best\_Solution \leftarrow Edge\_Labels$
end if
if $Level < n$ **then**
    $X \leftarrow \{x | x \in \{A, C, G, T\}, Count(Hits\_Pset[Level + 1] == x) > 0\}$
    $X_{open} \leftarrow \{IUPAC(\beta) | \beta \in powerset(X) - \emptyset\}$
    for $\beta \in X_{open}$ **do**
        $Hits\_Pset\_Child(\beta) \leftarrow Compute\_Hitset(Hits\_Pset, \beta)$
        $Hits\_Nset\_Child(\beta) \leftarrow Compute\_Hitset(Hits\_Nset, \beta)$
        $PNbrOnes(\beta) \leftarrow$ number of 1s in $Hits\_Pset\_Child(\beta)$
        if not $Bounded(PNbrOnes(\beta), Size\_Pset, Size\_Nset, Best\_Score)$ **then**
            $Best\_Solution \leftarrow$ **Traverse_IUPAC(**n**,** $Level + 1$**,** $Best\_Score$**,** $Best\_Solution$**,** $concat(Edge\_Labels, \beta)$**,** $Hits\_Pset\_Child((\beta)$**,** $Hits\_Nset\_Child((\beta)$**,** $Size\_Pset$**,** $Size\_Nset$**)**
        end if
    end for
end if
return $Best\_Solution$

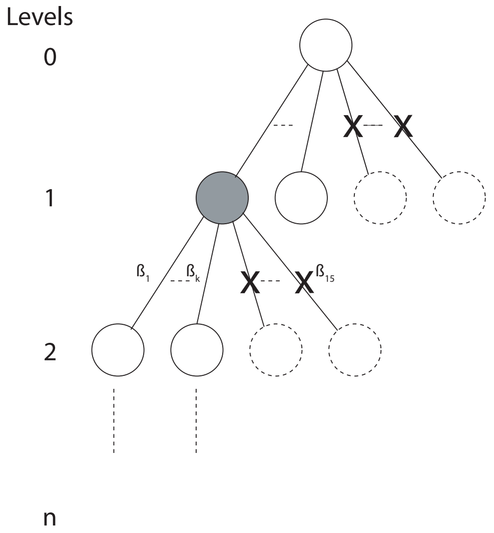

Figure 1: Depth-first search of IUPAC motifs.

that matches the first positions of a motif need not match an extension of the motif. We have therefore used a trivial lower bound of zero on FP (line 22). Some weak bounds on FP will often be possible to find, but it will require complicated and computationally expensive calculations with small effects on pruning.

Projection to next level: Base symbols not appearing in the next position of any positive instances of the current node can not improve motif score. So, it suffices to consider only symbols appearing at least once in the next position of such instances, as well as their combinations. This is exploited in line 16 and 17, resulting in reduced branching factor.

Pre-computation: Bit-strings are pre-computed for each non-degenerate/degenerate IUPAC symbol at each motif position (for the set of positive and negative substrings separately). Each bit in the bit-strings represents whether a respective instance matches a specific symbol in a position. This makes the computation in line 19 and 20 very efficient.

Reducing bit-string counting: We first consider each non-degenerate $\beta \in X_{open}$ in the loop at line 18 and count the 1s in $Hits\_Pset\_Child(\beta)$. For degenerate $\beta \in X_{open}$ there is no need to do bit-string counts, it suffices to sum the counts computed for constituents of β. This results in efficient counting in line 21.
